# Depressive symptoms and heart rate variability in perinatal women: A narrative review

**DOI:** 10.1111/jjns.12650

**Published:** 2025-01-28

**Authors:** Taeko Unno, Hisayo Okayama

**Affiliations:** ^1^ Graduate Course of Midwifery Kyoto Koka Women's University Kyoto Japan; ^2^ Institute of Medicine University of Tsukuba Ibaraki Japan

**Keywords:** anxiety, depressive symptoms, heart rate variability, perinatal period, stress

## Abstract

**Aim:**

This study aims to review research on heart rate variability and psychiatric symptoms in perinatal women and explains how heart rate variability can be useful in preventing depressive symptoms in perinatal women.

**Methods:**

Data were collected from PubMed, CINAHL, PsycINFO, and Google Scholar. The literature search encompassed articles published until July 2024, with the inclusion criteria targeting studies on women within 1 year postpartum, starting from the gestation period. Further, articles exploring this population that discussed the relationship between anxiety, depression, stress, and heart rate variability were selected. The exclusion criterion was studies that confirmed a correlation between stressors and heart rate variability.

**Results:**

We identified 36 relevant articles. The results demonstrated that, since 2022, research has been conducted using smartwatches, smartphones, and so on. The effectiveness of using heart rate variability has been confirmed, particularly in studies linking it to depression. However, some studies lacked controls during measurements. Intervention studies demonstrated the effectiveness of heart rate variability biofeedback.

**Conclusions:**

This is the first review to investigate the relationship between psychiatric symptoms and heart rate variability in perinatal women. Understanding and using the characteristics of heart rate variability may lead to the detection of psychiatric symptoms in perinatal women and to self‐care among women.

## 1 INTRODUCTION

Pregnancy and childbirth are important life events for women, but they can also be stressful. Especially in the postpartum period, it is easy for women to become stressed owing to changes in marital roles, child‐rearing, and lack of sleep. Women have difficulties recognizing that they are experiencing postpartum depression, and even when they are aware, they may be reluctant to seek help (Cacciola & Psouni, 2020; Sorsa et al., 2021).

Postpartum depression occurs in about 20% of women and can lead to suicidal thoughts, problematic mother–infant bonding, and possible cognitive impairment in children (Ikeda & Kamibeppu, 2013; Saharoy et al., 2023; Xiao et al., 2022). As postpartum depression occurs when stress responses, such as anxiety and depression, are elevated (Mariotti, 2015), there is a need to detect and respond to stress responses as early as possible. Additionally, perinatal psychological symptoms, such as anxiety, depression, and stress, are interrelated; and prenatal anxiety and depression are predictors of postpartum depression (Cheng et al., 2021; Obrochta et al., 2020; Wilcox et al., 2021). Therefore, it is important to understand mental health during the perinatal period. Currently, in Japan, women in the postpartum period are screened with the Edinburgh Postnatal Depression Scale (EPDS) during one‐month postpartum checkups and home visits (Ministry of Health, Labour and Welfare, 2023). However, these subjective reports may not fully capture the subject's experience of mood improvement or deterioration (Hobbs et al., 2021). Additionally, we believe that other methods are necessary to enable women to objectively identify their own psychological symptoms so that they may practice better self‐care.

Heart rate variability (HRV) is an objective method of assessing stress responses, such as anxiety and depressive symptoms, as well as anxiety and depression disorders. HRV is an electrocardiogram marker that reflects the activity of the sympathetic and vagus nerve components of the autonomic nervous system (ANS), located in the sinus node of the heart. The advantage of using this measurement is that it is noninvasive and can be used even when the patient is unconscious. Previous studies have reported associations between questionnaire scores (self‐reports) and HRV (an objective indicator) in stress responses, such as anxiety and depressive symptoms, as well as in severe anxiety disorders and major depressive disorder (Chang et al., 2013; Ham et al., 2023). Additionally, one review has examined the effectiveness of interventions using HRV indicators in patients with depression (Chen et al., 2022). However, little is known about the relationship between psychological symptoms and HRV in the perinatal period. Therefore, the purpose of this review is to clarify the current state and issues of research on HRV and psychiatric symptoms in perinatal women and to clarify how HRV can be useful in preventing depressive symptoms in perinatal women.

## 2 METHODS

### 2.1 Research design

This study used a narrative review design.

### 2.2 Literature search process

Literature was searched using the keywords “pregnancy,” “postpartum,” “antenatal,” “perinatal,” “anxiety,” “depression,” “stress,” “heart rate variability,” and “autonomic nerve” through PubMed (1966–July 2024), Cumulative Index to Nursing and Allied Health Literature (CINAHL; 2001–July 2024), and PsycInfo (1950–July 2024). Postpartum depression is interrelated with depression and stress and includes anxiety symptoms (Cheng et al., 2021; Wisner et al., 2013). Thus, we determined it is best to use the search string “(“Pregnancy” OR “Postpartum”) AND (“Depression” OR “Anxiety” OR “Stress”) AND (“Heart Rate Variability” OR “Autonomic Nerve”).” The search was also conducted by changing “(‘pregnancy’ or ‘postpartum’)” to “(‘antenatal’ or ‘perinatal’).”

Subsequently, a search was performed on Google Scholar. Cross‐referencing was performed from the reference lists of identified publications. The inclusion criteria were as follows: (1) literature on pregnant and puerperal women (within one‐year postpartum); and (2) literature on the relationship between HRV and anxiety, depression, and stress. The exclusion criterion was (1) studies that confirmed a correlation between stressors and HRV. We reviewed the titles, abstracts, and text of the extracted literature. We selected articles that met our study objectives. The resulting citations were exported to Microsoft Excel and manually reviewed to identify relevant publications.

### 2.3 Definition of terms

HRV is the periodic fluctuation of heart rate intervals (R‐R intervals). In this study, HRV is defined as “the means to find out the state of the ANS. The variation between heartbeat is low in sympathetic activation and high in parasympathetic mode” (Tiwari et al., 2021, p. 4). Assessments were performed with linear measurements, standard deviation of normal‐to‐normal intervals (SDNN), and root mean square of successive R‐R interval differences (RMSSD) in the time domain; and total power (TP), absolute power of the high‐frequency band (HF), and ratio of LF‐to‐HF power (LF/HF) in the frequency domain. Regarding nonlinear measurements, Poincaré plot standard deviation perpendicular to the line of identity (SD1) and Poincaré plot standard deviation along the line of identity (SD2) were used (Table 1).

**TABLE 1:** Heart rate variability time‐domain and frequency‐domain measures.

Parameter	Unit	Description
SDNN	ms	Standard deviation of normal‐to‐normal intervals
RMSSD	ms	Root mean square of successive R‐R interval differences
TP	ms^2^	Total power (distribution of R‐R; 0.004–0.4 Hz)
LF	ms^2^	Absolute power of the low‐frequency band (0.04–0.15 Hz)
HF	ms^2^	Absolute power of the high‐frequency band (0.15–0.4 Hz)
LF/HF	%	Ratio of LF‐to‐HF power
SD1	ms	Poincaré plot standard deviation perpendicular to the line of identity
SD2	ms	Poincaré plot standard deviation along the line of identity

Abbreviation: HRV, heart rate variability.

The entire frequency spectrum was assessed using TP (similar to SDNN); the sympathetic and parasympathetic nervous system activities were evaluated using SDNN and SD2; and the parasympathetic nervous system activity was evaluated using RMSSD, HF, and SD1 as indicators. LF/HF represents the overall balance between the sympathetic and parasympathetic nervous systems: a high value is considered an indicator of sympathetic dominance, and a low value is an indicator of parasympathetic dominance (Shaffer & Ginsberg, 2017; Task Force of European Society of Cardiology North American Society of Pacing Electrophysiology, 1996). The absolute power of the low‐frequency band (LF) was excluded in this study because of the inconsistency of its use: it has been used as an index of sympathetic (Heathers, 2014) or parasympathetic nervous system activity (Shaffer & Ginsberg, 2017).

Additionally, heart rate variability biofeedback (HRVBF)—a noninvasive therapy training—is defined as a means of increasing HRV (Lehrer et al., 2000).

## 3 RESULTS

### 3.1 Literature search results

**FIGURE 1:**
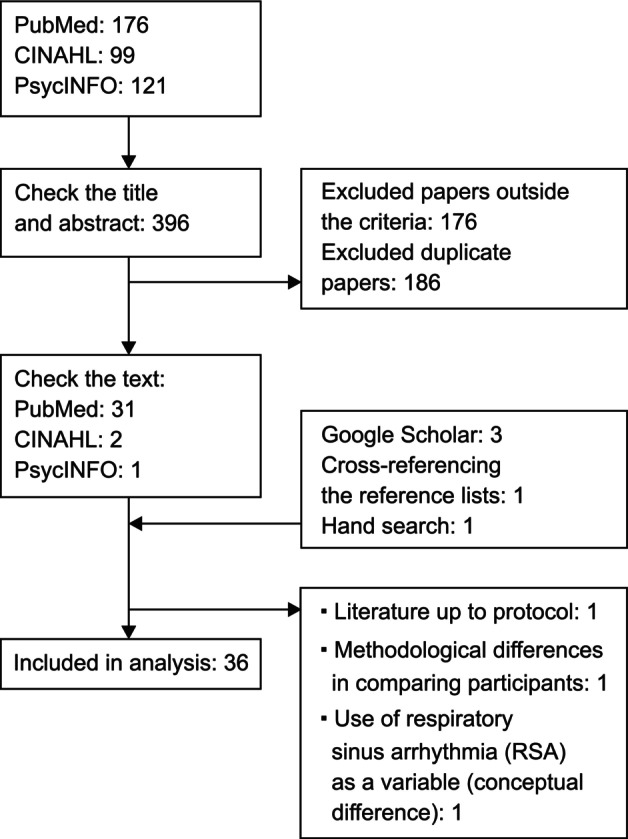
Literature extraction process. CINAHL, Cumulative Index to Nursing and Allied Health Literature.

A PubMed search using the specified search string yielded 176 articles, of which 31 were selected based on their titles and abstracts. The CINAHL search yielded 99 articles, and 2 were selected. PsycINFO yielded 121 articles, and 1 article was selected. Three articles were found on Google Scholar. One article was identified through cross‐referencing reference lists. One article was found through manual search. The texts of these 39 selected articles were checked, and 36 articles were included in this review. Three were excluded for employing a protocol design (*n* = 1), including respiratory sinus arrhythmia as a variable (*n* = 1), and using differences in comparison attributes (*n* = 1). The extraction process is shown in Figure 1.

### 3.2 Summary of the literature covered

Among the 36 articles, there were 8 cross‐sectional studies, 13 longitudinal studies, 13 intervention studies, and 2 review articles. Most measurements were taken while participants were resting, during load test, or during interventions, but five studies involved longer‐term measurements while participants were moving freely (Hayase & Shimada, 2018; Niela‐Vilen et al., 2023; Rouleau et al., 2016; Shea et al., 2008; Teckenberg‐Jansson et al., 2019). The devices used to evaluate HRV included electrocardiograms, photoplethysmography (PPG) monitors, and magnetocardiography. The PPG measurements have been conducted using a smartwatch (Niela‐Vilen et al., 2023), a smartphone camera (Singh Solorzano et al., 2022), a sensor bracelet (Bossung et al., 2023), or a ring (Balsam et al., 2023). Although it lacks a built‐in PPG, a wristwatch‐type heart rate sensor (cardio‐frequency meter) was also used previously (Ribeiro et al., 2018).

The publication years of the literature were as follows: 2005–2010, three articles; 2011–2015, seven articles; 2016–2020, 13 articles; and 2021–2024, 13 articles. In the articles of this review, the EPDS, the State‐Trait Anxiety Inventory, and the Perceived Stress Scale were the most common measures of depression, anxiety, and stress, respectively. All studies had reliable and valid scales except for two (Bossung et al., 2023; Niela‐Vilen et al., 2023).

### 3.3 Relationship between stress response and disease

We first examined studies that investigated the relationship between psychological symptoms and HRV assessed at the same time. Thirteen articles, including reviews, were identified (Table 2).

**TABLE 2:** Relationship between stress response and disease assessed during the same time in 13 articles.

Author and year of publication	Country	Sample	Measurement condition	HRV measurement tools and time	Scale	Results
Bossung et al. (2023)	Switzerland	23 healthy pregnant women	Sleep	Range: 8–25 weeks (average of 15 weeks) PPG (sensor bracelet)	Likert scale of eight emotions (including anxiety and stress)	Only the association with stress remained significant, with a positive association between SDNN and frequent feelings of stress; however, there was a negative association with the time interaction term
Brown et al. (2021)	United States	78 healthy perinatal women (exclusion criteria were diagnosis of a fetal anomaly or a major immunological condition of the mother)	No description	A small ambulatory monitor ECG for 5 min	CES‐D	No significant correlation
Herbell (2019)	United States	82 healthy pregnant women	After a pause; in sitting position	ECG for 5 min	CESD‐R	No significant correlation
Lee and Tae‐hee (2007)	South Korea	33 healthy postpartum women	After a pause; in sitting position	ECG for 5 min	EPDS	The group with depression symptoms had lower SDNN, HF, and TP
Mizuno et al. (2017)	Japan	65 healthy pregnant women	After a pause; in lateral position	ECG for 5 min	STAI	The group with anxiety had lower HF
Niela‐Vilen et al. (2023)	Finland	62 healthy perinatal women including with histories of preterm births or late miscarriages	Limitless	Smartwatch equipped with PPG; approximately 10 months	Subjective stress level 0–100	No relationship
Redpath et al. (2019)	United States	Literature				Increased vulnerability to PMADs is associated with reduced HRV.
Rouleau et al. (2016)	Canada	287 pregnant women; excluded were those with heart disease and hypertension at the time of enrollment	Limitless	A small ambulatory monitor ECG for 24 h	EPDS	HF was negatively correlated with depression symptoms
Shah et al. (2020)	India	172 pregnant women without clinical depression	After a pause; in supine position	ECG for 10 min	EPDS, PSS	SDNN, RMSSD, TP, and HF were negatively correlated with depression symptoms and stress. LF/HF was positively correlated with depression symptoms and stress
Shea et al. (2008)	Canada	81 pregnant women, including with a diagnosis of depression	Limitless	ECG for 24 h	EPDS, STAI MINI	The depressed group had lower SDNN. LF/HF was associated with depression (EPDS) during sleep time. Anxiety was not associated with any of the HRV measures.
Shimizu et al. (2015)	Japan	65 healthy postpartum women	After a pause; in sitting position	PPG (finger) for 3 min	EPDS	No significant correlation
Washio et al. (2020)	Japan	36 healthy perinatal women	After a pause; in supine position	PPG (finger) for 3–5 min	Oxytocin	Multiparous perinatal women; oxytocin correlated positively with HF, TP
Zöllkau et al. (2019)	Germany	141 healthy pregnant women	In supine position	MCG for 30 min	DASS‐42	TP was negatively correlated with stress

Abbreviations: CES‐D, Center for Epidemiologic Studies Depression Scale; CESD‐R, Center for Epidemiologic Studies Depression Scale–Revised; DASS‐42, Depression Anxiety Stress Scale–42; ECG, electrocardiogram; EPDS, Edinburgh Postnatal Depression Scale; HF, absolute power of the high‐frequency band; HRV, heart rate variability; LF/HF, ratio of LF‐to‐HF power; MCG, multifunction cardiogram; MINI, Mini‐International Neuropsychiatric Interview; PMADs, perinatal mood and anxiety disorders; PPG, photoplethysmography; PSS, Perceived Stress Scale; RMSSD, root mean square of successive R‐R interval differences; SDNN, standard deviation of normal‐to‐normal intervals; STAI, State‐Trait Anxiety Inventory; TP, total power.

#### 3.3.1 Association with anxiety

Four studies identified associations with anxiety (Bossung et al., 2023; Mizuno et al., 2017; Shea et al., 2008; Zöllkau et al., 2019), including three in healthy individuals and one involving a diagnosis of depression. Only one study found a link between HRV and anxiety symptoms in healthy women at rest (Mizuno et al., 2017). In the literature comparing groups with high and low levels of anxiety symptoms, a significant decrease in HF was observed in pregnant women with high levels of anxiety symptoms (Mizuno et al., 2017).

#### 3.3.2 Association with depression

Seven studies identified associations with depression, including six in healthy individuals (Brown et al., 2021; Herbell, 2019; Lee & Tae‐hee, 2007; Rouleau et al., 2016; Shah et al., 2020; Shimizu et al., 2015) and one involving a diagnosis of depression (Shea et al., 2008). Three studies observed an association between depression and HRV (Lee & Tae‐hee, 2007; Rouleau et al., 2016; Shah et al., 2020). Some studies found that depression was negatively correlated with HRV (SDNN, RMSSD, TP, and HF) and positively correlated with LF/HF (Rouleau et al., 2016; Shah et al., 2020). In a study comparing groups with high and low levels of depression symptoms, a significant decrease in SDNN, HF, and TP was observed in postpartum women with a high level of depression symptoms (Lee & Tae‐hee, 2007).

Three studies found no association during the measurement (Brown et al., 2021; Herbell, 2019; Shimizu et al., 2015). One study reported that the HRV parameters were evaluated only in the SDNN of the time domain and were not the best measure of comparison (Herbell, 2019). Other studies did not describe the controls they used, such as the timing of feeding in postpartum surveys (Brown et al., 2021; Shimizu et al., 2015). Specifically, since the parasympathetic nervous system becomes dominant when breastfeeding directly, it is necessary to avoid measuring HRV for 30 min after breastfeeding (Grewen & Light, 2011; Mezzacappa et al., 2005; Ohmura et al., 2023).

A study of pregnant women diagnosed with depression found that they had lower SDNN in the depressed group (Shea et al., 2008). This study was associated with LF/HF and depressive symptoms (EPDS) during sleep. However, no correlation was found with daytime or 24‐h LF/HF (Shea et al., 2008).

#### 3.3.3 Association with anxiety/depression

Oxytocin is thought to have antidepressant/anxiolytic effects in postpartum women. In multiparous perinatal women, oxytocin correlated positively with HF and TP (Washio et al., 2020). A review of the microbiome, including the perinatal ANS, reported that reduced parasympathetic activity increases vulnerability to perinatal mood and anxiety disorders (Redpath et al., 2019). However, in this study, scant literature linked perinatal HRV to perinatal mood and anxiety disorders.

#### 3.3.4 Association with stress

Four studies identified associations with stress in healthy women. Three studies revealed associations between HRV and stress in resting and healthy women (Bossung et al., 2023; Shah et al., 2020; Zöllkau et al., 2019). They found that stress was negatively correlated with HRV (SDNN, RMSSD, TP, and HF) and positively correlated with LF/HF (Shah et al., 2020; Zöllkau et al., 2019). Bossung et al. (2023) found that the reduction in SDNN was more pronounced in women who frequently experienced stress. One study (Niela‐Vilen et al., 2023) did not find an association, possibly due to the lack of impact of pregnancy on current stress in participants with a history of preterm and late miscarriages. That study used longitudinal data obtained using a smartwatch. However, the results were unclear because a single measurement item was used for the questionnaire, and the validity of the measurement scale was not verified. The possibility of participants being affected by motion artifacts was also noted (Niela‐Vilen et al., 2023). Motion artifacts are special types of noise content arising because of physical activity. In previous research, the PPG signal changed significantly during motion, which makes the reported PPG useless for direct HR calculation (Ismail et al., 2021).

### 3.4 Immediate evaluation using the load test

The relationship between stress response and disease after the load test was investigated in five papers as shown in Table 3. An association between anxiety and HRV was found in Braeken et al.'s (2015) study, where load testing was conducted on women without medical conditions. In women with medical conditions, some studies found connections between HRV and a past or current diagnosis of anxiety disorder, obsessive‐compulsive disorder, childhood trauma events, depression, and adjustment disorder (Braeken et al., 2013; Kimmel et al., 2021; Riddle et al., 2023; Shinba et al., 2024).

**TABLE 3:** Relationship between stress response and disease after the load test in five articles.

Author and year of publication	Country	Sample	Measurement condition	HRV measurement tools	Scale	Results
Braeken et al. (2013)	Netherlands	56 pregnant women, including with history of anxiety disorders.	The arithmetic problem task	Three‐electrode ECG for 25 min (1st)	MINI (2nd), STAI (1st)	Resolved anxiety disorders group had lower HF and RMSSD.
Braeken et al. (2015)	Netherlands	157 healthy pregnant women	Alternating task and relaxation; arithmetic problem task	ECG	STAI	The HF of pregnant women with anxiety declined more significantly upon second presentation of the task.
Kimmel et al. (2021)	Switzerland	126 pregnant women, including criteria for any psychiatric diagnosis	The working memory task	PPG (finger)	MINI, EPDS, STAI‐Trait	Those with several types of past or current anxiety disorders, greater trait anxiety, or greater exposure to childhood traumatic events had significantly different HRV than the others. Lower HF was found for women who met the criteria for a history of obsessive‐compulsive disorder.
Riddle et al. (2023)	United States	49 pregnant women, including those with anxiety disorders	Two psychological stressor tasks	Three‐electrode ECG	STAI, PSS, SCID, and clinician interview	The RMSSD between the task and recovery in the anxiety group decreased. In all women, RMSSD was negatively correlated with stress.
Shinba et al. (2024)	Japan	45 postpartum women including AJD and PPD diseases	The random number generation task	Wearable ECG device	Mental health diagnosis according to DSM‐5	PPD showed a lack of adequate HRV regulation in response to the task load, accompanying a high LF/HF score in the rest state. Women with AJD exhibited high HF and reduced LF/HF during the after state.

Abbreviations: AJD, adjustment disorder; DSM‐5, Diagnostic and Statistical Manual of Mental Disorders–5; ECG, electrocardiogram; EPDS, Edinburgh Postnatal Depression Scale; HF, absolute power of the high‐frequency band, HRV, heart rate variability, LF/HF, ratio of LF‐to‐HF power; MINI, Mini‐International Neuropsychiatric Interview; PPD, postpartum depression; PPG, photoplethysmography; PSS, Perceived Stress Scale; RMSSD, root mean square of successive R‐R interval differences; SCID, structured clinical interview for DSM; STAI, State‐Trait Anxiety Inventory.

### 3.5 Prediction of stress responses

The relationship between HRV and stress response at different pregnancy periods was investigated in four studies, as shown in Table 4. Three previous studies revealed that pregnancy HRV predicts postpartum depression (Eriksson et al., 2024; Kishan et al., 2023; Singh Solorzano et al., 2022). However, one paper (Parisi et al., 2024) found no association with depression. Parenting stress at 4 months postpartum was associated with HRV at 6 months postpartum (Parisi et al., 2024).

**TABLE 4:** Relationship between heart rate variability and stress response at different assessment times in four articles.

Author and year of publication	Country	Sample	Measurement condition	HRV measurement tools and time period	Scale (period)	Results
Eriksson et al. (2024)	Switzerland	122 perinatal women, including with depression and with anxiety	The working memory task	38 weeks pregnant, PPG (finger) for 10 min	BAI, EPDS (6 weeks postpartum)	Group comparisons indicated that lower pregnancy HRV was associated with depressive or anxious symptomatology. HRV indices were predictors in a combined model with background and pregnancy variables.
Kishan et al. (2023)	India	35 healthy perinatal women	After a pause; in supine position	ECG for 5 min (>32 weeks gestation)	EPDS (6–8 weeks postpartum)	A significant positive correlation was found between LF/HF and EPDS.
Parisi et al. (2024)	Norway	106 healthy perinatal women	The babies were placed on their parents' laps, facing a person from the research group performing the screening	ECG for 15–20 min (6 months postpartum)	PSI (4 months postpartum), EPDS (16–22 weeks gestation, 4 months postpartum)	Depressive symptoms did not predict decline in RMSSD. Higher (overall) perceived parenting stress was associated with lower RMSSD.
Singh Solorzano et al. (2022)	Italy	135 perinatal women without mental illness	After a pause; in sitting position	ECG and PPG; smartphone application for 2 min (2nd)	EPDS (1 month postpartum)	Decreased RMSSD during pregnancy predicts postpartum depression.

Abbreviations: BAI, Beck Anxiety Inventory; ECG, electrocardiogram; EPDS, Edinburgh Postnatal Depression Scale; HF, absolute power of the high‐frequency band; HRV, heart rate variability; LF/HF, ratio of LF‐to‐HF power; PPG, photoplethysmography; PSI, Parenting Stress Index; RMSSD, root mean square of successive R‐R interval differences.

### 3.6 Heart rate variability‐related intervention studies

Intervention studies assessed with HRV and feedback intervention studies using HRV techniques were reported in 14 papers, as shown in Table 5. The effectiveness of yoga and music therapy interventions using HRV measurements to reduce anxiety, depression, and stress has been demonstrated (Hayase & Shimada, 2018; Li & Dong, 2012; Ribeiro et al., 2018; Satyapriya et al., 2009 ; Teckenberg‐Jansson et al., 2019). Further, three mindfulness‐based intervention studies were identified (Balsam et al., 2023; Muthukrishnan et al., 2016; Rådmark et al., 2023), one of which included a control group with a Lamaze intervention (Rådmark et al., 2023). In Balsam et al.'s (2023) study, RSSMD decreased after the intervention, a result that differed from their hypothesis. In the authors' report, it is unclear whether the lack of change in HRV was a natural occurrence during pregnancy or a sample size problem (Balsam et al., 2023; Rådmark et al., 2023).

**TABLE 5:** Heart rate variability‐related intervention studies in 14 articles.

Author and year of publication	Country	Sample, period before intervention	Intervention methods		HRV measurement tools and time, measurement condition	Scale	Results
			Type	Method			
Hayase and Shimada (2018)	Japan	91 healthy pregnant women 20–23 pregnancy week	Yoga	Intervention group: 60 min once a week in the hospital yoga room and 15 min daily at home (until delivery) Control group: Not attending yoga classes	ECG was recorded for 24 consecutive hours	PSS	HF was significantly higher in the intervention group than in the control group.
Satyapriya et al. (2009)	India	90 healthy pregnant women 18–20 pregnancy week		Intervention group: Successively practiced two modules of integrated yoga, specifically designed. Control group: Practiced standard prenatal exercises	ECG Intervention group: Continuously for 5 min before, 10 min during, and 5 min after the deep relaxation technique period control group: Supine rest	PSS	In intervention group, the HF increased, and the LF/HF were concomitantly reduced (significant difference between groups). No correlation between stress and HRV.
Li and Dong. (2012)	China	60 women who are scheduled for a Cesarean section	Music	Intervention group: Listened to slow‐rhythm music for 30 min in quiet surroundings. Control group: Relax and rest for 30 min	ECG Details unknown	SAS	The mean HRV, as measured by the LF/HF, was significantly less after the music intervention. Moreover, the mean HF value was significantly increased and the mean anxiety score was significantly decreased after the music intervention.
Ribeiro et al. (2018)	Brazil	46 postpartum women with premature infants		Intervention group: Individually, and lasting from 30 to 45 min (average of seven sessions) Control group: No mention	Wristwatch‐style heart rate sensor, 12 min All women were measured in silence	BAI, BDI	In comparisons between the groups, the intervention group showed an improvement in anxiety and depression scores and SDNN, RMSSD, and SD1 Correlations were found between depression and RMSSD and SD1 for the intervention group.
Teckenberg‐Jansson et al. (2019)	Finland	102 high‐risk pregnant women Second trimester		Intervention group: 30 min for three consecutive days Control group: No music therapy	Two‐electrode ECG was worn for 3 days. Intervention group: 20–30 min of music therapy, control group: Rested	PSS, STAI‐Trait	SD2 increased significantly more in the intervention group than in the control group.
Balsam et al. (2023)	United States	20 healthy pregnant women (no control group) 10–32 pregnancy week	Mindfulness	Practice with an application twice daily during the month‐long trial (range of 530–1050 min)	PPG; Ring Style (Oura ring). During sleep from 4 days before intervention to 1 month after intervention	PSS GAD‐7 PRAS	Reductions were found in stress, anxiety, and pregnancy anxiety. Contrary to hypothesis, RMSSD decreased.
Muthukrishnan et al. (2016)	India	74 healthy pregnant women 17–18 pregnancy week		Intervention group: Administered two sessions per week for 5 weeks Control group: Received usual obstetric care	ECG for 3 min (standing), followed by 20 min (stand → supine position)	PSS	There was a significant decrease in stress scores and a significant increase in HRV in the intervention group compared with the control group.
Rådmark et al. (2023)	Sweden	60 healthy pregnant women 20–25 pregnancy week		Intervention group: Nine 3‐h long weekly sessions Control group: Lamaze program	Three electrodes ECG for 5 min After a pause; in supine position	EPDS, PSS	The intervention had no effect on measures of HRV. Improvement in depression, stress in both groups.
Beckham et al. (2013)	Switzerland	15 women admitted to the perinatal psychiatric hospitalization (no control group) Currently pregnant or within 1 year of delivery	HRVBF	Two sessions ranging in length from 30 min to an hour	No HRV measurement	STAI, EPDS	Improvement in anxiety.
Herbell and Zauszniewski (2019)	United States	Literature					Peripartum women who completed HRVBF reported a reduction in depression and stress compared with participants who did not receive HRVBF.
Kudo et al. (2014)	Japan	55 healthy postpartum women postpartum Day 4		Intervention group: Performed daily Control group: No HRVBF	PPG (finger) for 5 min. After a pause; in supine position	EPDS	There was a significant decrease in depression in the intervention group; after adjusting, there were significant increases in SDNN in the intervention group compared with the control group.
Siepmann et al. (2014)	Germany	48 women at risk of premature birth 24–32 pregnancy week		Intervention group: Six sessions of over 2 weeks Control group: Assigned to control sessions	No HRV measurement	BDI, STAI, TICS, Brief Symptom Inventory Symptomatic distress	In the intervention group, the perception of chronic stress was decreased, but there was no change in the control group. No difference in anxiety, stress, and depressive symptoms, between the groups.
Van der Zwan et al. (2019)	Netherlands	50 healthy women including pregnant women 19 average pregnancy week		Intervention group: Five weekly meetings (60–90 min each) Control group: After the intervention group ended, the control group also underwent HRVBF	No HRV measurement	DASS‐21 PSS HADS	Results indicated a beneficial effect on anxiety complaints for pregnant women. No significant effect was found for the other stress‐related complaints.
Van Dijk et al. (2024)	Netherlands	156 perinatal women without mental illness. <28 pregnancy week and currently smoking		Intervention group: 8‐week using a mobile application Control group: Received a link plain psycho‐education etc. on pregnancy	No HRV measurement	PSS	Stress reduced in both conditions.

Abbreviations: BAI, Beck Anxiety Inventory; BDI, Beck Depression Inventory; DASS‐21, Depression Anxiety Stress Scale‐21; ECG, electrocardiogram; EPDS, Edinburgh Postnatal Depression; GAD‐7, General Anxiety Disorder‐7; HADS, Hospital Anxiety and Depression Scale; HF, absolute power of the high‐frequency band; HRV, heart rate variability; HRVBF, heart rate variability biofeedback; LF/HF, ratio of LF‐to‐HF power; PPG, photoplethysmography; PRAS, Pregnancy‐Related Anxiety Scale; PSS, Perceived Stress Scale; RMSSD, root mean square of successive R‐R interval differences; SDNN, standard deviation of normal‐to‐normal intervals; SAS, self‐rating scale for anxiety, SD1, Poincaré plot standard deviation perpendicular to the line of identity; SD2, Poincaré plot standard deviation along the line of identity; SDNN, standard deviation of normal‐to‐normal intervals; STAI, State‐Trait Anxiety Inventory; TICS, The Trier Inventory for the Assessment of Chronic Stress.

HRVBF therapy was also used during the perinatal period. HRVBF, a slow breathing technique (approximately 6 breaths/min), could improve HRV parameters and vagal tone (Burlacu et al., 2021). Two studies (Kudo et al., 2014; Siepmann et al., 2014) included in the systematic review reported that perinatal women who completed HRVBF therapy experienced reduced depression and stress compared with participants who did not receive HRVBF therapy (Herbell & Zauszniewski, 2019). No information was reported regarding the effectiveness of HRVBF therapy for psychological stress in the first and early second trimesters of pregnancy (Herbell & Zauszniewski, 2019). However, published papers have confirmed the benefits of HRVBF therapy for women from early pregnancy through to the postpartum period (Beckham et al., 2013; Van der Zwan et al., 2019; Van Dijk et al., 2024).

## 4 DISCUSSION

### 4.1 Research trends and recommendations for anxiety, depression, stress, and HRV during the perinatal period

While studies on HRV have been conducted since the early 1940s, the importance of HRV has evolved, and studies using wireless wearable devices have been conducted since the 2010s (Ishaque et al., 2021). Thirty‐six articles were extracted for this review, of which four studies (Balsam et al., 2023; Bossung et al., 2023; Niela‐Vilen et al., 2023; Singh Solorzano et al., 2022) conducted in around 2022 used smartwatches, smartphones, sensor bracelets, or rings. Research measuring HRV by placing a finger on the camera of a smartphone equipped with PPG has begun (Moya‐Ramon et al., 2022) and is expected to increase, given the simplicity and low cost of the measurement. Wearable devices are noninvasive, low‐cost, easy to operate, and useful for evaluating activities of daily living and determining health‐related information (Castaneda et al., 2018; Vijayan et al., 2021). A groundbreaking study with pregnant women (Bossung et al., 2023) used the Ava Sensor Bracelet (Ava AG), a wristwatch‐type device registered with the U.S. Food and Drug Administration, during sleep for an average of 15 weeks. Given that most women in the perinatal period live at home rather than in a hospital, in the future, wearable devices may be useful for perinatal women's self‐care.

This review on perinatal psychiatric symptoms and HRV has included survey, load test, and intervention studies. Thirteen studies examined the relationship between stress responses and illness at the same assessment times. Specifically, HRV was associated with anxiety in one out of four studies, depression in four out of seven studies, and stress in three out of four studies, with depression showing an association. Furthermore, this review found that HRV during pregnancy was associated with predicting postpartum depression in three cases. A previous study (Carnevali et al., 2018) also reported a link between depressive symptoms and HRV. Therefore, early detection of depressive symptoms is possible. All papers for the immediate evaluation of the load test showed positive results; however, the number of studies was too small to identify any trends. A review (Cheng et al., 2022) of research on HRV in non‐perinatal patients with anxiety disorders found no alterations in HRV reactivity (all reactivity data, data on physiological challenge, and psychological challenge). Two types of HRV intervention studies were included: those that clarify the intervention effects using HRV indices and those that use the principles of HRV in the intervention. Few intervention studies used HRV as an index. Nevertheless, this review identified the effectiveness of interventions using HRVBF. Furthermore, previous studies with non‐perinatal populations have demonstrated the effectiveness of HRVBF as an intervention (Lehrer et al., 2020; Pizzoli et al., 2021). HRVBF is freely available and could benefit perinatal women in their daily lives.

### 4.2 Issues and recommendations for research on perinatal anxiety, depression, stress, and HRV

The first challenge in the literature identified by this review was participant selection. Brown et al. (2021) suggested that the absence of participants with high depression scores might explain their unclear results. If the participant is less depressed, anxious, or stressed, this may not be reflected in the results. Prior studies have consistently demonstrated an association with HRV in participants with a clinical diagnosis of depression or anxiety disorder. However, participants with nonclinical, self‐reported depressive and anxious symptoms showed a variety of findings (Paniccia et al., 2017). This suggests that the intensification of symptoms may affect the results of the association. In addition, the effect of parity on symptoms is known (Nakamura et al., 2020) and might have influenced the results. Therefore, research needs to be conducted considering the attributes of the participants.

Next, pregnancy stages must be considered. Balsam et al. (2023) found results that differed from their hypothesis, which they explained by the effects of pregnancy. As pregnancy progresses, the sympathetic nervous system becomes more dominant (Kolovetsiou‐Kreiner et al., 2018). Surveys conducted 3 months after giving birth have shown that women's autonomy nervous systems return to their prepregnancy states (Sarhaddi et al., 2022). If the effects of the intervention are observed over a long period of time during pregnancy, the parasympathetic nervous system might not have become dominant due to the influence of pregnancy and not due to the intervention. Therefore, when examining perinatal women's HRV, it is necessary to consider whether it is due to pregnancy.

Additional issues were identified regarding self‐administered questionnaires and HRV indicators. Herbell (2019) reported that no association was observed because HRV was not the best predictor; this is because depressive symptoms were assessed only using SDNN in the time domain, which is an HRV parameter. SDNN is an indicator of the parasympathetic nervous system when measured at rest for short periods of time (Shaffer & Ginsberg, 2017); SDNN decreases in the presence of depression (Koch et al., 2019). SDNN is also considered an indicator of physiological resilience to stress (Kim et al., 2018). However, HF and RMSSD are parasympathetic indices that decrease when one is stressed (Sheridan et al., 2021); further, RMSSD is a better indicator than HF because it is unaffected by respiration (Pham et al., 2021). Since RMSSD is more strongly influenced by the parasympathetic nervous system compared with SDNN (Pham et al., 2021), it is desirable to use RMSSD as well to clarify the association. Additionally, although self‐administered questionnaires can easily allow researchers to assess participants' subjective symptoms, stress is not experienced only through conscious perception (Epel et al., 2018). When combined with an objective evaluation, it may be possible to understand the underlying condition. Therefore, when investigating stress, both subjective and objective evaluation data should be comprehensively analyzed.

Regarding issues with measurement methods, some studies (Brown et al., 2021; Shimizu et al., 2015) did not describe premeasurement controls. Short‐term HRV analysis is suitable for outpatient care and short‐term patient monitoring, with the advantage of receiving instantaneous results. However, the measurement of HRV must consider potential confounders, such as age, sex, medical history, posture, diurnal variability, diet, and exercise (Laborde et al., 2017; Voss et al., 2015). Moreover, women who breastfeed their babies have parasympathetic nervous system dominance due to breastfeeding (Mezzacappa et al., 2005). Therefore, obtaining more accurate data by standardizing conditions, such as breastfeeding, would be desirable among participants prior to data collection. Li et al. (2019), who examined participants in conditions in which they were free to move around, found no association during the day or over a 24‐h period. Long‐term HRV analysis allows us to obtain the status of women going through a dynamic daily routine, but the data collected from the more active hours are noisy (Li et al., 2019). Consequently, comparing participants is challenging. Therefore, when assessing women in the perinatal period, it is advisable to evaluate them at comparable times, such as during rest or sleep.

## 5 CONCLUSIONS

HRV tends to decrease in individuals with depressive symptoms; furthermore, HRVBF has been effective in treating psychological symptoms. Therefore, HRV may be useful for screening women and HRVBF for promoting relaxation by enhancing parasympathetic nervous system activity.

Currently, there is limited literature on the relationship between HRV and psychiatric symptoms during the perinatal period; nevertheless, an increasing number of studies use devices such as smartwatches to measure HRV over the long term. When assessing HRV, we believe that considering a woman's pregnancy stage and the method of measuring HRV will enable mental health support that is more closely aligned with women's daily lives.

## AUTHOR CONTRIBUTIONS

Taeko Unno and Hisayo Okayama contributed to the conception and design of this review study. Taeko Unno contributed to the screening and study selection, and to the collation, summary, and reporting of results. Taeko Unno drafted the manuscript; Hisayo Okayama critically reviewed the manuscript, supervised the entire process. All authors read and approved the final manuscript.

## CONFLICT OF INTEREST STATEMENT

The authors declare that there is no conflict of interest.
